# Nutritional Quality and Socio-Ecological Benefits of Mare Milk Produced under Grazing Management

**DOI:** 10.3390/foods13091412

**Published:** 2024-05-04

**Authors:** Ana Blanco-Doval, Luis Javier R. Barron, Noelia Aldai

**Affiliations:** Lactiker Research Group, Department of Pharmacy and Food Sciences, Faculty of Pharmacy, University of the Basque Country (UPV/EHU), 01006 Vitoria-Gasteiz, Spain; ana.blancod@ehu.eus (A.B.-D.); luisjavier.rbarron@ehu.eus (L.J.R.B.)

**Keywords:** horse, milk nutritional value, grazing management, socio-ecological benefits

## Abstract

This review discusses the scientific evidence that supports the nutritional value of mare milk and how its properties are essentially achieved when mares are managed under grazing conditions. Mare milk’s similarity with the chemical composition of human milk makes this food and its derived products not only suitable for human consumption but also an interesting food regarding human health. The contribution of horse breeding under grazing management to other socio-ecological benefits generated by equine farms is also highlighted. Both the high added value of mare milk and the socio-ecological benefits derived from pasture-based systems could be explored to improve the performance of equine farms located in arid and semi-arid areas or in regions with moderately harsh environmental conditions as equids have a strong adaptation capacity.

## 1. Introduction

The most primitive evidence of horse domestication and mare milk production is attributed to the Botai culture, from the Eurasian steppe of northern Kazakhstan, dated around 3500 years before the Christian era [1]. There is also evidence of domesticated horses from the late Shang Dynasty in China (the second century before the Christian era [2]). Currently, the production and consumption of mare milk is concentrated in Mongolia, Russian Buryatia and Kalmykia, Bashkortostan, Kazakhstan, Kyrgyzstan, Tajikistan, Uzbekistan, northern China, Tibet, and Xinjiang [2,3], where about 8% of pastoral milk production comes from mares [4]. In Mongolia, pastoral nomads traditionally consume meat during the cold season and milk products during the warm season. Among these, airag (fermented mare milk) is the most popular traditional food, but it is not evenly produced throughout Mongolia, presumably due to cultural and ethnicity factors [5].

During the last few decades, mare milk consumption has extended to Europe, mainly to Belarus, Ukraine, France, Belgium, Germany, the Netherlands, Norway, Austria, Hungary and Bulgaria [2,6]. While cow milk accounts for 83% of world milk production, only less than 0.1% corresponds to mare milk [7]. However, there are no official data about mare milk production and consumption worldwide, and it is not easy to estimate as it is assumed that most of it is self-consumed in households. Uniacke-Lowe and Fox [8] estimated a production of 1–1.3 million L of equine milk per year in Europe, whereas, according to Minjigdorj and Austbø [9], 8 million L of mare milk is annually produced in Mongolia. In the past decade, it was estimated that 30 million people consumed mare milk regularly worldwide [10]. However, real data are uncertain.

Mare milk productivity is an important matter of concern due to two main factors. First, horse breeds have generally not been selected for improved milk production (except for some Asian breeds), although any horse breed can become a dairy herd as long as mares accept being milked [2]. Consideration of the integration of mare milk production into future selection schemes for some breeds could be an option. Second, due to mare mammary biology, milk production is low. These two factors can result in low milk yields, which could be limiting depending on the characteristics of the farm and the objectives associated with milk production.

Horses are a seasonal polyoestrous species that impregnate between May and October. With gestation periods of approximately 11 months [11], lactation occurs between spring and autumn, lasting 5 to 6 months. Milking usually starts 20 days after parturition to allow the foal to suckle the colostrum and the initial milk, as it is necessary for its adequate development and growth [10]. The aforementioned small udder capacity of mares (approximately 2.0–2.5 L in heavy horse breeds [12,13]) can be, according to Turabayev et al. [14], compensated by more efficient milk production. Approximately 75–85% of the milk produced corresponds to alveolar milk, with a very low cisternal capacity [10], and the udder can be refilled in 1.5–2 h, allowing foals to suckle every 0.75–2 h [12], but also permitting several milkings per day. Overall, mare milk production is estimated at an average of 2.5 kg per 100 kg of live weight per day in heavy breeds [10], peaking somewhere between the first and the third month of the lactation period [13]. According to Mongolian data under extensive systems, 580–640 mL of milk can be obtained in each milking [15] and, after multiple interventions, up to 1.7–5.0 L of milk per day can be obtained from light breeds, which is two to three times less compared to heavy horse breeds [14,15,16]. It has been described that Mongolian extensive farming systems can allow 5–8 milkings per day during summer [5,15,16]. More milkings result in lower yields at each milking but a higher total yield per day [14].

Milk ejection is a consequence of oxytocin release, which, in mares, is often insufficient to completely empty the alveoli. As a result, it is common to have some residual milk, which is rich in fat, from one milking/suckling in the next one [13,17]. Mare–foal physical contact during milking [10,17,18] and oxytocin injections can effectively increase the fat content of milk [17]. Another strategy to maintain milk productivity is to supplement a mare’s diet with compound feeds when grass availability decreases since they contain protein, starch and micronutrients that can increase milk production. Daily energy and nitrogen requirements for milk production in early lactation (until 3 months) are 2.0–2.3 and 3.0–3.5 times the maintenance requirements, respectively. In late lactation (4 months and over), energy and nitrogen requirements decrease to between 1.6 and 2.4 times the maintenance requirements [10].

Another factor influencing milk yield is the milking method. According to Caroprese et al. [18], in Murgese mares, machine-milking is more effective than hand-milking in collecting residual milk, resulting in higher milk yields (7.7 kg and 4.9 kg of milk, respectively) and higher fat content, with the added benefit of saving effort and time for the farmer. Conditions are commonly set at a vacuum level of 45–50 kPa and 120–160 pulsations per minute [13,14,18]; slower pulsations can result in incomplete udder emptying, whereas faster pulsations can create anxiety for mares and inflammatory edema in their nipples [14]. Maximum milk production yields are reached at the age of 7–15 years for mares [19], and multiparous mares produce higher milk yields than primiparous ones [20].

As mentioned before, mare milk is a traditional dairy product in some regions of Asia and Russia, and it is mainly consumed as koumiss (also called airag or chigee in Mongolia and Inner Mongolia), a fermented alcoholic beverage very popular in western Asia, Mongolia, northern China and Russia [5,21]. In Asia, mare milk (either raw or fermented) has extensively been used as a medicine to treat tuberculosis, chronic hepatitis, peptic ulcers and heartburn [3,21]. In Europe, mare milk is consumed raw or frozen, as well as freeze-dried in the form of capsules, pills or powder packages, and it can also be found as an ingredient in cosmetics [3]. The European Union establishes that raw milk can be destined for human consumption as long as it comes from healthy animals, following the corresponding health and quality verifications [22]. Considering that mares are unlikely to suffer from mastitis [21] and that the biological quality of their milk is usually acceptable [10], raw mare milk should be easily marketable in Europe, avoiding the alteration and loss of thermosensitive components. However, it is a perishable product and, as indicated below, the high polyunsaturated fatty acid (PUFA) content might suffer from oxidation processes, which is why its transformation might improve conservation.

Mare milk is an interesting dairy product due to its high nutritional value, as will be discussed later in this work. Specifically, it has lower casein and higher whey protein proportions than ruminant milk and higher lactose contents (Table 1). In addition, the monogastric digestive system of horses and direct absorption of dietary lipids makes mare milk rich in unsaturated fatty acids, particularly forage-derived n-3 PUFA, compared to ruminant milk (Table 2). This makes mare milk an interesting food in terms of human health.

Currently, mare milk production is not high compared to other dairy domestic species, but its commercialization as a quality product obtained under extensive breeding systems could provide an added-value product. Considering this, the present review explores the potential benefits of implementing pasture-based mare milk production in current equine farms that produce horses for human consumption (mainly producing horse meat). As discussed below, due to its beneficial health properties, the production of mare milk in Europe could progressively increase by reaching a niche market within the functional foods market and particularly addressing those consumers with special dietary requirements, such as children and the elderly, and/or by including mare milk into other products such as cosmetics.

## 2. Nutritional Value of Mare Milk

Mare milk production under extensive farming could contribute to the provisioning of high-quality food to society. In this section, the characteristics of mare milk are described and compared to those of cattle (large frame) and sheep (small frame) species. The composition of cow, mare and ewe milk—including lipids, proteins and other compounds—is detailed in Table 1.

### 2.1. Source of Lipids

Bovine and ovine milk lipids consist mainly of triglycerides (98%), whereas mare milk has been reported to have a higher content of phospholipids (5%), sterols (4.5%), glycolipids (1%), and free fatty acids [11]. Mare milk contains a broad diversity of lipids [58] and a lower cholesterol content compared to cow and ewe milk [6].

The total fatty acid (FA) composition of extensively reared mare milk differs considerably from grazing ruminant milk (Table 2), which is primarily related to digestive physiology, and, as in other milk-producing species, it can be affected by the feeding and lactation stage [36]. Mare milk is considerably richer in PUFA than ruminant milk [3,4,5,6], which is of interest for human nutrition. This is because, unlike ruminants, horses’ digestive fermentation takes place in the caecum–colon compartment, allowing for the earlier absorption of dietary FAs (before microbial biohydrogenation). Moreover, the continuous secretion of biliary salts and high concentration of pancreatic lipases optimize the digestion of dietary lipids in the small intestine. In addition, it seems that high galactolipase activity in the horse digestion tract benefits the absorption of pasture lipids—rich in 18:3n-3 (α-linolenic acid, LNA)—which are present as galactolipids in a high proportion [59]. Galactolipase activity has been partially attributed to pancreatic lipase-related protein 2, which is present in horses [60,61]. This enzyme could be responsible for the efficient liberation of PUFAs from galactolipids and their high deposition in horse tissues and fluids, especially when managed under grazing [59,62].

Overall, grass-based mare milk is characterized by 18–31% PUFA and 43–58% saturated FA (SFA) (Table 2). The major FAs are palmitic (16:0), oleic (9*c*-18:1), linoleic (LA; 18:2n-6) and LNA (18:3n-3) acids; LA and LNA proportions can vary depending on the lipid composition of grasslands [37,45]. Compared to ewe and cow milk, the stearic (18:0) acid content is particularly low while the capric (10:0), lauric (12:0) and palmitoleic (9*c*-16:1) acid contents are higher in mare milk. On a percentage basis, mare milk is considerably rich in n-3 PUFAs [63], even though long-chain PUFA contents, such as eicosapentaenoic (20:5n-3), docosapentaenoic (22:5n-3) and docosahexaenoic (22:6n-3) acids, are low (Table 2). Taking into account equid digestive physiology, the content of both *trans*-18:1 and conjugated LA isomers is low, and their minor presence is an indicator of their limited microbial metabolism in the intestine [59,64].

As with other mammalian species [63], the FA composition of mare milk varies with breed [65,66], the age of the mare, foaling number [39] and, mainly, lactation [37,39,65,66] and nutrition [66,67,68]. At the beginning of lactation, when a high metabolic load is required, FAs derived from adipose tissue mobilization predominate in ewe and cow milk, while dietary FAs are relevant later [69,70]. Horses, however, are less able to mobilize body reserves and compensate for their high voluntary intake [71], which explains why dietary FAs are so abundant in mare milk.

Concerning the influence of diet, ruminants fed on pasture contain more unsaturated FAs than those fed with concentrates, particularly LNA, rumenic acid (9*c*,11*t*-18:2) and some biohydrogenation products derived from PUFA. This is due to a higher availability of LNA from forages [45,72]. Overall, very few studies have analyzed the effect of diet on mare milk FA composition. A recent lipidomic study comparing milk from mares fed pasture, silage or stover found that grazing mares’ milk contained significantly more lipids related to PUFA and lipid digestion and absorption metabolic pathways [68]. Otherwise, knowledge regarding the composition of pasture-based mare milk lipids relies on studies performed under extensive mountain conditions in the Mongolian steppe (>1000 m of altitude [40]) and Kyrgyz Republic (2200 m approx. [73]), along with others where the lactation period occurred during the grazing period (i.e., from spring to autumn [36,37,38,39]). But, unfortunately, differences in the experimental conditions related to the scientific literature have resulted in high variability of reported milk FA composition. Indeed, some of the reported variations in fat content and FA composition may be related to differences in extraction, separation and identification methods where, in some cases, the chromatographic columns and conditions used might not have been sufficient to separate potentially co-eluting FAs [74,75]. As a whole, it can be concluded that grazing management systems provide milk with a higher PUFA (n-3 and/or n-6 FAs derived from botanical species) in most mammalian species, but particularly in equids characterized by monogastric physiology and low efficiency of mobilizing body reserves.

In relation to the distribution of lipids in the milk matrix, fat forms dispersed globules of a smaller size (2–3 µm diameter; Table 1) and different layer structure compared to ewe and cow milk [47,76]. Mare milk fat globules are covered with three layers; the external layer is comprised of high-molecular-weight glycoproteins coated with a branched oligosaccharide structure. This layer might enhance the binding of lipases to the fat globules, and both the size and the cover composition have been reported to facilitate the digestion of milk lipids by humans [76].

### 2.2. Source of Proteins

The total protein content of mare milk is lower than that of ruminant milk, especially compared to ewe milk. One of the most remarkable differences is that the whey protein content is about 20% and casein is about 80% in cow and ewe milk, whereas it is 45% and 55%, respectively, in mare milk (Table 1). In fact, cow milk is commonly named a “casein type milk”, while mare milk is considered an “albumin type milk” [3].

Mare milk contains less β-lactoglobulin but more α-lactalbumin, immunoglobulins (Ig) and lysozymes compared to bovine and ovine milk (Table 1). Mare milk contains similar quantities of α-lactalbumin and β-lactoglobulin, whereas β-lactoglobulin is the predominant whey protein in most ruminants [77]. Three genetic variants of α-lactalbumin (A, B and C) and two β-lactoglobulin isomers (I and II) are present in mare milk [77]. β-lactoglobulin has a monomeric form in horse milk, whereas it is a dimer in ruminant milk [13], and the equine isomer II has been described to be structurally similar to the human retinol-binding protein [2]. The biological function of equine β-lactoglobulin is not clear, but, unlike ruminant β-lactoglobulin, it is known to lack the ability to bind FAs. In fact, serum albumins are the only proteins with the ability to bind FAs in mare and human milk, whereas, as mentioned, ruminant β-lactoglobulin can perform that task too [2,77].

Lactoferrin, lysozyme and Igs, together with the lactoperoxidase system, have been reported to contribute to the antimicrobial effect of milk [27,77]. Mare milk is particularly rich in lactoferrin and lysozyme, except for comparable contents of lactoferrin in ewe milk (Table 1). Lactoferrin is an iron-binding enzyme that has shown antimicrobial, antiviral, anti-inflammatory, anti-oxidative and immunomodulatory properties, and it is an enhancer of the growth of specific probiotic strains [77,78]. The iron-binding capacity of equine lactoferrin, related to an antibacterial mechanism, is similar to human and greater than bovine lactoferrin [77].

On the other hand, equine lysozyme is particularly interesting due to its calcium-binding capacity, which results from the integration of active sites from α-lactalbumins (calcium-binding proteins) while maintaining the enzymatic activity of non-calcium-binding lysozymes [77]. Since equine lysozyme is resistant to acid and proteolysis, it might reach the human intestine practically intact [6]. In addition to its antimicrobial effect, antiviral, anti-inflammatory, immunomodulatory, antifungal and anti-carcinogenic properties have been attributed to lysozyme [3,78]. So, considering the bioactive properties of these two enzymes—lysozyme and lactoferrin—the high abundance in mare milk is an attractive feature from product functionality and human nutrition perspectives.

The abundance of Igs in mare milk is considerably high (Table 1), but, interestingly, its profile differs from that of other species. IgG predominates in bovine colostrum and milk, whereas, in human colostrum and milk, IgA is the main Ig. Conversely, IgG predominates in mare colostrum, but IgA predominates in mare milk. This happens because, contrary to humans, the transfer of IgG to the fetus via utero is inefficient in ruminants and equids, so it needs to occur via colostrum [77]. Igs in milk have antibacterial, anti-inflammatory and immunomodulatory properties [78], and their main function is to protect the newborn [3].

In terms of caseins, these are conformed in micellar structures in which calcium is transported, and mare casein micelles have a wider diameter than cow or ewe micelles (Table 1). In this sense, the bigger mare milk micelles imply a low micelle surface-to-milk volume ratio. The casein profile of mare milk is very similar to that of human milk. While ruminant milk contains high and similar concentrations of αs1- and β-caseins, mare and human milk are principally composed of β-casein, and αs1-concentrations are significantly lower. Both human and equine milk contain notably low αs2- and κ-casein levels (Table 1 [6,41]). The only and always glycosylated casein in equine milk is κ-casein, which is located on the micellar surface. Its main function is to stabilize the casein micelle, and the high glycosylation makes it more resistant to hydrolysis by chymosin than bovine κ-casein. This fact, together with the low protein content and casein/whey protein ratio, as indicated before, makes mare milk unsuitable for cheesemaking, with low cheese yields due to difficulties in renneting and curd forming. From a human nutrition perspective, low contents of casein, αs-casein and β-lactoglobulin and high contents of lysozymes in mare milk resemble the protein composition of human milk [77].

Mare milk is often promoted as low-allergenic milk that could be an adequate substitute for infant formula for infants with a cow milk protein allergy. This low allergenicity has been speculated to derive from a high susceptibility of equine β-lactoglobulin to gastrointestinal digestion and the low content of some allergenic proteins (such as αs2-casein) in milk [3]. However, few studies have addressed mare milk protein allergenicity using either in silico, in vitro or animal models or clinical studies [79,80,81]. Moreover, up-to-date limited studies on the digestibility of equine proteins (including β-lactoglobulin) have been performed [82,83], so firm conclusions cannot be drawn. However, these studies suggest that mare milk does indeed present low allergenicity in individuals with a cow milk protein allergy, so this is a topic worth studying.

### 2.3. The Source of Other Components

Considering that extensive breeding systems have an impact on the lipid composition of mare milk, it is expected that other high-nutritional-value compounds, such as fat-soluble vitamins, are also affected. Vitamins A and E are the major fat-soluble vitamins present in mare milk [37], even though their content is lower than in milk from other mammal species (Table 1), probably due to a lower total fat content. Horses are known as “yellow fat animals” for their ability to absorb dietary β-carotene (precursor of retinol or vitamin A) and transfer it to tissues including milk [84]. Pasture is a great dietary source of carotenes for horses and, as a result, the seasonality and composition of the botanical species can influence the retinol status of mare serum, which might, at the same time, impact mare milk retinol content. In fact, unsupplemented mares on pasture can achieve similar serum retinol levels than mares kept indoors and supplemented with retinyl palmitate [85]. Other authors also found increased β-carotene levels in mares’ plasma once the grazing period started. Supplementation with β-carotene can increase its presence in mare milk, although it might not be reflected in the vitamin A content [86], probably due to metabolic regulation processes in the horse [28], resulting in an overall low vitamin A content in mare milk (Table 1). Overall, the content of α-tocopherol in mare milk is low (Table 1). However, two independent studies have demonstrated that α-tocopherol supplementation during pregnancy significantly affects mare milk and colostrum α-tocopherol contents [87,88], demonstrating the positive effect of dietary α-tocopherol intake in mare milk vitamin E levels. Despite all this, and due to limitations in the current knowledge regarding the contribution of pasture-based diets to fat-soluble vitamin contents in mare milk, further research is essential.

Other minor compounds of high nutritional value present in mare milk are water-soluble vitamins and minerals (Table 1). In this regard, vitamins of the B group have a different metabolism in ruminants and monogastrics. In ruminants, rumen microorganisms can synthetize most of them, while monogastrics depend on their diet to fulfill their vitamin requirements. Thus, the influence of feeding reported in ruminant studies is not applicable to horses [89]. Overall, milk water-soluble vitamins are more affected by diet than fat-soluble vitamins, although factors affecting water-soluble vitamin concentration in mare milk as well as their composition under extensive systems have not been studied in depth. The scientific literature shows that mare milk is particularly poor in vitamins B2 and B9, but, together with ewe milk, it contains higher amounts of water-soluble vitamins compared to cow milk, mainly due to a higher ascorbic acid (vitamin C) content [6] (Table 1).

Equid milk, together with ewe milk, is among the mammal milk with the lowest total mineral content. Despite the low calcium and phosphorus concentration in mare milk (Table 1), its higher calcium/phosphorus ratio compared to cow and ewe milk makes it more favorable for human nutrition [63]. Other trace elements have been found in lower or similar amounts than in cow [42,90] and ewe milk produced under extensive systems [48,49,50], except for copper, which is 1.5–2.5 times higher in mare compared to cow [42] and ewe milk [49,50].

Regarding carbohydrates, equine milk is richer in lactose (6.5 %) than most other mammalian milk (Table 1) [63]. Oligosaccharides, mainly linked to the surface of the external glycoprotein layer in the fat globule membrane [2], are of lower concentration and diversity in bovine and ovine milk [91], whereas mare colostrum and milk provide a great oligosaccharide quantity and structural diversity [92,93]. Some of the oligosaccharides seem to be specific to mares [93]; however, this may depend on the breed or genetics [92].

## 3. Socio-Ecological Benefits of Horse Production under Grazing Management

Extensive equine production is slowly gaining relevance in line with environmental alternatives that attempt to move away from industrial agriculture. Equines need to be introduced in international political debates, especially those dealing with the agro-ecological transition of animal production systems and their contribution to sustainable development goals. At the European Union level, we need to better understand and use equine green assets so that the equine sector can contribute to an agro-ecological transition and regional development [94]. In this regard, the new legislation of the Common Agricultural Policy for the period 2023–2027 includes the professionals of the equine sector as eligible for some subsidies under particular conditions [95].

European Union policies promote innovations in sustainable production systems within the current societal systems [94]. Livestock sustainability assessments mainly consider their economic and environmental contributions and often leave out other services such as product quality, rural vitality and cultural heritage. In fact, only food provisioning has a clear market price (monetary metrics), while other goods/services from livestock activity do not have such a tangible economic value, but should be quantified in some way and, of course, considered in order to assess the positive impact of pastoral equine farming systems [96]. In line with Ryschawy et al. [97] and Dumont et al. [98], we consider that there is a need to assess and value all the services that livestock provide to society and their interrelations. In this section, after discussing how horse characteristics allow them to adapt to graze vegetation from pastures and the animal welfare of mares managed under extensive grazing, we analyze the environmental benefits of grazing horses and the consequences for rural vitality and cultural heritage.

### 3.1. Equine Adaptation to Extensive Management in Less Favoured Areas

According to the Food and Agriculture Organization (FAO) of the United Nations [99], 41% of the global land surface can only be utilized for food production by autochthonous domestic herbivores that are able to transform vegetation from grasslands and shrublands into food. In the context of climate change, local breeds that are well adapted to harsh environmental conditions will possibly be the key to maintaining food production in less favored regions, particularly in Mediterranean areas [100]. With adverse hydrological conditions and strong human impacts, these Mediterranean areas are particularly susceptible to desertification [101], and locally adapted equids, which are part of the traditional grazing system, could play an important role in nature conservation and the prevention of land degradation [100].

Equines are non-ruminant herbivores. They have a mono-chambered stomach where non-fermentative digestion takes place (foregut), while the fermentation process takes place in the large intestine or caecum–colon compartment (hindgut fermentation) [102,103]. Horses strongly select bites dominated by grasses [104,105,106] and generally use dicotyledons to a lower extent compared to cattle because they are less able to detoxify their secondary metabolites [104,107]. Horses graze longer than ruminants (15 h per day on average vs. 8 h per day in ruminants) and their food intake is less constrained by the particle size of digesta. They are thus able to ingest larger amounts of forages, especially roughages, than ruminants [104,108,109].

In spring, when sward biomass is abundant and of high quality, horses preferentially graze in habitats consisting of improved pastures or grasslands, where they benefit from high-quality grasses [109,110,111,112,113,114,115,116]. Doing this, they select a diet containing more protein and/or energy and less fiber than available vegetation [109,116]. In these grasslands, horses mainly use tall vegetative sward areas that are more accessible until these become mature [117]. Then, they switch to high-quality short swards (below 8 cm) and avoid areas of tall mature grass where they concentrate their droppings [117,118]. This behavior was explained as an anti-parasitic strategy [119], although some more recent works indicate that the nutritional characteristics of grass also play an important role in the choice of feeding sites [117,120,121]. The continuous exploitation of previously grazed patches by horses is a key mechanism through which they shape the structure of the whole plant community by creating stable patches of short swards within a matrix of tall vegetation contaminated with their feces [122]. In general, cattle are excluded from the shortest swards below 4 cm where their bite depth is limited [123] and, therefore, leave these areas [112], while horses can stay, taking advantage of their two sets of incisors [104]. In cases where sward availability in grasslands becomes limiting also for horses, they generally switch to areas of poorer nutritional quality, such as heathlands [109,112,113,116]. Horses can travel long distances to new feeding sites in order to satisfy their feeding requirements [102]. In heathlands, horses were observed to select highly nutritive legumes like gorse and avoid other woody species (heather and other shrubs) [110,113,114,115,116]. Overall, horses and lactating mares have shown good adaptation capacity in shrublands and heathlands due to their ability to select highly nutritive leaves, green stems, flowers and pods among woody plant material [109,110,116]. In contrast, horses are limited when mobilizing body reserves [71], and, therefore, milk production by mares could be compromised when the availability of green biomass becomes low [110] as their nutrient requirements are 75% higher than those of dry mares [11].

The long daily grazing time of horses allows them to compensate for their lower fiber digestion efficiency compared to ruminants so that they maintain their daily intake even in poor-quality environments [104,116]. Several works have proved that grazing horses achieve adequate growth performance and can maintain their body weight all along the grazing season [106,113,114,124]. Lactating mares of light breeds under unlimited herbage conditions were shown to maintain their daily intake, meet their dietary requirements and produce foals with satisfactory growth and conformation while relying only on herbage [125]. Mares were, however, underfed and fell short of their requirements when the daily herbage allowance was less than 39 kg of dry matter (DM) per mare per day [125] or when fed on heather-dominated heathlands (due to a lower preference for this type of vegetation) [114].

**Figure 1:**
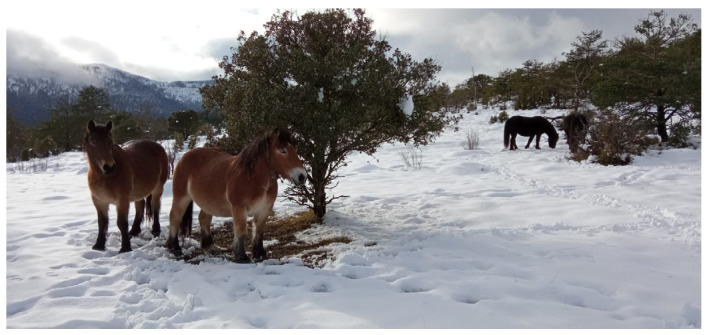
Basque mountain horses feeding in a mountain area in the Basque Country (Spain) in winter conditions.

Horses can adapt to extensive management during winter (Figure 1) [126,127] better than cattle [128]. As an example, Brinkmann et al. [126] showed that domestic Shetland ponies (*Equus ferus caballus*) were able to adapt their body temperature (subcutaneous temperature) and heart rate under harsh winter conditions, which is similar to the hypometabolism strategy of their wild ancestor the Przewalski horse (*Equus ferus przewalskii*, originally from the Mongolian steppe) [129]. Horses are able to limit their walking activity during winter as an energy-saving strategy, limiting most of their movement time to feeding and avoiding lying down [126]. Coat hair density also varies according to temperature, especially in free-ranging animals [130]. Among those facing extreme environmental conditions, horses of the Yakutian breed can be found in the northeastern part of the Russian Federation, where temperatures range from −60 °C in winter to 40 °C in summer [131]. People who emigrated there in the 13–15th centuries brought along already domesticated horse breeds that had developed additional physiological and morphological adaptations (i.e., improved hair density, fat accumulation and body size compaction) to maintain body temperature and survive under severe climatic conditions. Yakutian horses graze all year long, consuming vegetation even when it is buried under a thick layer of snow [132]. Horses are also able to adapt to semi-desert conditions [133] or short periods of water deprivation [134].

It is recognized that native breeds are better adapted to physiological, nutritional and disease stress and, hence, to living in natural wild conditions [96,130,135]. In the context of climate change, autochthonous horse breeds offer opportunities for sustainable agricultural production in less favored [135] and extensive areas [136] as they can survive extreme environmental conditions like prolonged droughts or severe snowing episodes that cause the death of most free-ranging livestock [137]. Moreover, horses are easy to manage and have been reported to face lower vulnerability to predation compared to other large herbivores [138].

### 3.2. Health and Welfare of Grazing Mares

Animal welfare is an indicator of sustainable livestock production systems [139]. According to the Animal Welfare Indicators (AWIN) Welfare Assessment Protocol for Horses, it has to do with adequate feeding and housing conditions and good health, but also with the expression of social and other behaviors, good human–animal relationships and positive emotional states of the animals [140]. Horses are defined as social animals since they naturally live in hierarchized groups and need social interactions with conspecifics [141]. In this sense, extensive livestock production systems where animals spend time outdoors, live through natural experiences and perform species-specific behavior are in line with the adequate practices reported, as long as the extreme environmental conditions (e.g., harsh climatic conditions in winter or food and water scarcity) are not harmful to the animals. It has been described that horses with access to paddocks show less stress and aggressive behavior, lower risk of injury, lower prevalence of stereotypies (repetitive and invariant behaviors) and, in general, better welfare and positive social behavior [142,143].

European legislation only compels animal welfare labeling for egg production, and welfare standards and labeling for other animal products are scarce. Simultaneously, consumers are not adequately informed about the management practices used for the foods they are consuming, such as dairy products. In response to this situation, some organisms and institutions at national and international levels have implemented their own labeling systems to encourage animal welfare on farms. Some examples are Freedom Food in the United Kingdom, Label Rouge in France, some foods under Protected Designations of Origin and Protected Geographical Indications [144] and the Welfair^TM^ certificate in Spain [145], which is based on the Welfare Quality^®^ and AWIN^®^ projects. None of these are related to equid milk or meat. Promisingly, the European Commission announced that harmonized labeling for sustainable food choices will be proposed within the Farm to Fork Strategy [146]. For now, the European Commission has established a sub-group within the European Union Platform on Animal Welfare dedicated to labeling [147]. There is also a European regulation that contemplates animal grazing with regard to organic production and labeling [148]. This regulation limits animal density on pastures and establishes minimum pasture-derived feed rates. It includes equids, but not the ones destined for milk production.

An important pillar of animal welfare is animal health. Good health valorization includes the absence of injuries, disease and pain. The use of antibiotics is an issue of concern in human health due to the development of resistant pathogens as well as the spread of antibiotic resistance genes through the food chain. Dairy cattle are usually administered antibiotics for the prevention and treatment of intra-mammary infections, mainly mastitis [149]. However, mastitis is uncommon in mares [21], so antibiotics as a prevention tool seem less needed. In organic dairy systems (in which extensive management systems are often included), the use of antibiotics as prophylactics or growth promoters is forbidden, and they are only accepted in extreme cases in which phytotherapeutic or other treatments are not effective. If animals are administered antibiotics or antiparasitic medication more than three times within a year (or more than one time in yearlings), they must stay under strict organic conditions for six months before their milk can be commercialized as organic again [148].

### 3.3. Environmental Impact of Grazing Equines

The maintenance of grassland-based systems is key for a number of ecosystem services (ES) related to the high natural value of landscapes, biodiversity conservation, and the preservation of soil and water quality [97,150]. Permanent grasslands have a key role in terms of carbon sequestration and, thus, in climate regulation and the maintenance of soil fertility [151]. The moderate intensification of nutrient-poor permanent grasslands, use of light grazing instead of intensive grazing and conversion of grass leys to grass–legume mixtures or permanent grasslands, also related to equine livestock, can increase carbon stocks [152].

It is known that grassland biodiversity can be compromised by both overgrazing and abandonment of pastoral lands [127,153,154,155,156]. Under moderate (approximately 660–675 kg of live weight/ha) to high (up to 1080 kg of live weight/ha) stocking rates, horse grazing preserves the structural heterogeneity of grasslands [106,157]. As indicated, horses create a grassland habitat made of short sward patches within a mosaic of tall vegetation. The inter-annual stability of these grazing patterns [122] benefits the co-existence of different animal and plant species with different environmental preferences, enhancing pasture biodiversity [106,157]. The grazing of equines influences pasture structure and composition differently compared to that of cattle, sheep or goats. Grazing impacts also depend on whether horses graze alone or mixed with ruminant species [109]. Under a moderate stocking rate, co-grazing horses and cattle would maximize botanical diversity compared to only horse grazing as cattle increase the diversity in tall vegetation areas that horses avoid [157]. Horses are also less selective than cattle or sheep on forbs, so their direct grazing impact on flowering plants and flower-visiting insects is smaller [158,159]. Consequently, horses have a strong potential to enhance pasture biodiversity [124,158,159,160], except when the stocking rate increases considerably and horse trampling decreases the abundance of flowering plants [161].

The high voluntary intake of roughage by horses controls competitive grasses, which maintains “open” pastures and enables the coexistence of many plant and animal species [127,159]. While horses seem less able than cattle to control woody plants under extensive conditions [114,127], they can reduce some species, especially gorse, through grazing and trampling [110,115,160,162] and enhance grassland species diversity through selective grazing [110,115,162]. The reduction in gorse accumulation also opens the canopy and facilitates the access of other grazers to expanding grassland areas [163]. Mixed grazing with cattle generally improves the control of shrubs, especially when sward availability decreases in grasslands [116,127]. These strategies to improve pasture quality in shrub-dominated areas permit the recovery of abandoned lands by increasing biodiversity and nutritive plants and soils, without compromising animal performance and health.

Herbivores also benefit from the dispersal of plant seeds, attached to their coat or through their digestive tract [164], especially if grazing occurs after flowering [165]. Moreover, moderate equid grazing also contributes to the accumulation of organic matter (animal wastes) and, hence, the improvement of soil quality and productivity. Horse dung and urine deposited directly on the soil act as a natural fertilizer via the accumulation of nutrients [166]. Irrespective of the animal species, feces consist of water, undigested fodder, residues, animal metabolites, microorganisms and microbial metabolites [167]. In horses, the nitrogen excreted in feces is made up of 85–95% protein (57% microbial protein and 43% endogenous protein). Horse feces only contain 5–8% ammonia nitrogen and are rich in phosphorus (organic and inorganic; 75–85 mg per kg of body weight) and other minerals such as calcium (90–100 mg per kg of body weight), magnesium (15–20 mg per kg of body weight), potassium (15–25 mg per kg of body weight) and sodium (8–30 mg per kg of body weight) [168]. Moreover, they contain small quantities of other elements like heavy metals, which are not assimilated by the animal following the ingestion of vegetable matter or soil. It is known that herbivore species play a key role in nutrient fluxes among plants–animals–soils and in soil fertility. In this sense, not only are the nutrient contents and their chemical form important but also the mass of these nutrients related to the number and quantity of droppings [167]. In saddle horses of 500 kg fed on green or dry fodder, the average daily production of feces is estimated at 8–9.5 g DM per kg of body weight. As previously indicated, among domestic herbivores, horses are known to maintain areas of short grass within plots by grazing and avoid areas of tall grass where they concentrate their droppings. This leads to phosphorus and potassium depletion in heavily grazed areas and the enrichment of latrines [168]. Repeated grazing and trampling in short grass areas have positive impacts, especially in environments that are initially saturated with an excess of organic matter. Soils benefit from the unclogging of the catabolic chain of organic matter and are improved in their nitrogen mineralization potential. Plants, however, are limited in their growth by their small leaf area index and the low potassium availability in these areas [168]. At a local scale, heavily grazed areas are composed of short vegetation with little standing biomass, and low but high-quality primary production (young leaves). Plants rebuild their reserves to a limited extent. The root system is poorly developed and often weakened by the small amount of assimilate allocated to it, most of which is used to reconstitute leaves. In these conditions, small, prostrate (stoloniferous species) or rosette species, favored by their grazing avoidance strategy, are selected. Conversely, the high biomass accumulation in areas avoided by horses is associated with a decrease in vegetation quality. The local nutrient enrichment stimulates primary production, leading to strong competition for light. In these areas, large, highly competitive or eutrophic species are selected. These plants present a ruderal strategy that enables them to develop rapidly in areas enriched with mineral elements and maintain their dominance by taking up space (large rosettes, broad and spreading leaves) and producing a large number of seeds [168].

When livestock is managed extensively, soil and water contamination with chemicals and other residues is limited, and water pollution by fecal elements is mitigated, although not totally eliminated [169]. In extensive systems, especially in mountain grasslands, no water for human consumption is used, and livestock consumes “green water”—the water fraction stored as soil moisture. In addition, animals that graze in pastures not only do not compete with human food production but also convert non-human-edible matter (grass and forages) into human-edible foods (meat and milk) [170].

Agricultural land abandonment leads to the invasion of highly flammable dead biomass and woody vegetation (i.e., shrubs, heather and gorse), increasing the prevalence of wildfires [112,116,160,171], which will probably be more frequent with worsening climate change [172]. Great problems after a fire are soil erosion [173,174] and biodiversity loss [110], in addition to the devastation of established fauna and flora and organic matter quality and quantity. For instance, some nutrients are affected, pollutants appear, soil microbiota is lost and water infiltration and soil water holding capacity are hindered [174]. This displays an imperative need for implementing prevention mechanisms [175]. Horses showed good potential to remove more herbaceous vegetation per unit of body weight than cattle and graze closer to the ground, as reported by Pardini et al. [176] in a study performed on firebreaks using sown botanical species and grazing horses.

Greenhouse gas emissions from livestock account for 14.5–18% of global emissions [158,177], while livestock enteric fermentation accounts for 33–39% of total methane emissions derived from agriculture [178]. Hindgut fermenter herbivores, such as equids, generate methane in the caecum–colon [103] but in much lower amounts than ruminant species in the rumen [179]. This may be explained by a shorter retention time in the hindgut than in the rumen, resulting in lower fermentative degradation of plant cell walls and an alternative non-methane-producing hydrogen sinking route (acetogenesis) occurring more actively in the horse hindgut [103]. The annual release of enteric methane by equids in France has been calculated to be 20,202 tons from a total of 975,000 animals, giving an emission number of 20.7 kg per animal per year. This value increases to 29.7 kg when calculated for lactating mares, representing just 34% of the value calculated for milking cows [168,180]. Some authors have proposed a shift from ruminants to monogastrics as an alternative to mitigate greenhouse gas emissions [181]. However, when referring to monogastrics, most authors include porcine and poultry, and only a few consider equids as a high-quality protein source. It is true that equids represent only 4 × 10^−3^ % of livestock for meat production in Europe [182] but, due to its high nutritional value, horse meat consumption is gaining some interest in several countries [59]. In addition, recent studies showed that there is a rising potential to increase the consumption of horse meat in European countries [183,184]. However, there are still strong emotional and cultural reasons preventing consumers from eating horse-derived foods [185].

### 3.4. Rural Vitality

Indicators of rural vitality (rural activation and development) are mainly related to the contribution of livestock to rural employment and stability. Rural employment concurrently entails population growth and fixation but also requires investments in other social aspects such as social and institutional organizations; educational, technological and social services; infrastructures; gender perspectives; etc., which impact the overall life quality of the rural population [186,187,188]. As reported by Cooper et al. [189], to maintain traditional agricultural systems and associated knowledge (traditional education), an adequate population density is needed in rural and less favored areas. However, rural abandonment and consequent depopulation are occurring in Europe [190], and significantly lower economic development and employment rates are happening in rural compared to more urbanized communities [191]. This is driven by different factors such as unfavorable biophysical conditions of agricultural lands and socioeconomic factors of farms and farmers that are poorly supported by public administration policies and national and international markets (depending on the European region) [192].

Agricultural areas comprise almost half of the European territory, and one-third of them correspond to permanent grasslands and meadows [193]. On average, the percentage of workers dedicated to agriculture in the European Union has decreased to less than half in the last 30 years [194], although the farming sector still employs 4 million people in Europe, of which 25% are related to the dairy industry [195]. In relation to the equine sector, this provides the equivalent of 400,000 full-time jobs in Europe [196] and, in France, almost 100 different professions have been related, directly or indirectly, to the equine industry [197].

One of the strategies proposed to reinforce rural development and vitality is to make economies thrive through the diversification of economic activities [190]. Other diversification activities such as leisure, sport or equestrian activities are a source of direct economic benefit in local areas [198]. Therefore, new sources of profit, such as the production of non-traditional agricultural products (i.e., mare milk), could also be decisive in increasing farm incomes [199], especially in those equine farms exclusively dedicated to horse meat production. Moreover, grazing practices allow for lower investments in feeding and can somehow compensate for the low inputs of equine breeding [200]. Keeping in mind that a considerable proportion of citizens aim to recover relationships with the traditional rural life and are more attracted to natural environments for different reasons (i.e., close contact with nature, recreation and sports, spiritual development, cultural experiences, landscape, because they look for traditional education or want to escape from pandemic-related risk and isolation) [156,201], horse breeding for milk production could be another option that could be complemented by other activities.

### 3.5. Cultural Heritage

In this subsection, indicators such as heritage landscape, agrotourism, heritage animal products and genetic resources are considered [97]. European landscapes have been shaped through a long period of agricultural activities such as livestock grazing. Characterized by their heterogeneity, they are considered part of the cultural identity of many regions [189]. Landscape heterogeneity is related to biodiversity [202] and aesthetic value [156,201,203], which are directly compromised by land abandonment and the associated encroachment and homogenization of shrubs and forests [204] and urbanization, but also by agricultural intensification [155,205] and other economic activities such as intense touristic and recreational exploitation [171,205]. Mosaic-rich landscapes, open meadows and permanent grasslands with traditional agricultural buildings, farming systems and the presence of moderate livestock grazing are the elements of hedonic preference that most attract tourism [156,201,203].

Focusing on equines, cultural heritage and identity are usually linked to autochthonous horse breeds [135], which are largely being substituted with more productive international breeds for agricultural purposes [135,206], resulting in a loss of genetic biodiversity. Taking into account the overall genetic pool, horses are recognized as the second mammalian species (after rabbits) with the highest global at-risk breed percentage (33%) [207]. Therefore, autochthonous horse breeds need special attention, protection and support.

Equines can be connected with cultural heritage in many different ways such as part of leisure and sports activities (equitation), gastronomy or because they have traditionally been work animals [208], being an important part of the identity of regions. For instance, a number of equine-related affairs are a good example of “Intangible Cultural Heritage”, recognized by the United Nations Educational, Scientific and Cultural Organization [209].

Farms and households based on pastoralism usually work with autochthonous and well-adapted breeds [135], being a tool for gene preservation (primarily of those associated with the adaptation to free-ranging conditions) and promoting cultural heritage and the protection of traditional education and knowledge [136,204]. In this sense, it is recognized that extensive equine farming can contribute to the conservation of traditional and/or cultural landscapes [171,201], given that it creates a mosaic landscape due to species-specific grazing preferences.

## 4. Conclusions

Mongolia, Kazakhstan and other Asian countries are the main producers of mare milk in the world, to the point of considering it a traditional dairy food. Even though mare milk consumption has extended to several European countries, its production in Europe is still scarce, first of all, because equids are not great milk producers (due to the udder structure), and, second, because the production of mare milk and its derived products, as well as its benefits for human health, have not been deeply studied or adequately promoted.

From what is known today, any horse breed could be a potential milk producer under sustainable livestock management systems in order to produce milk with high nutritional value. Horses are part of the culture in many European and worldwide regions. Their incorporation into local farming could help protect local and regional breeds as well as provide other ecosystem services that would benefit both the farmer and society. This strategy could be of interest in arid and semi-arid regions (i.e., Mediterranean areas) or areas with moderately harsh environmental conditions (i.e., northern Europe, mountainous regions) as equids have good adaptation capacity.

In this respect, equine farmers need public authorities at different levels to recognize the ecosystem services provided in terms of not only food provisioning but also the maintenance of mountains and other natural areas (including biodiversity, landscape, soil and water quality, and the prevention of forest fires), the support of rural economies, and the preservation of the genetic pool of local horse breeds and cultural heritage.

## Figures and Tables

**Table 1:** Comparison of the content of lipids, proteins and other compounds among cow, mare and ewe milk.

	Cow	Mare	Ewe
Lipid fraction			
Fat content (%)	3.4–5.0 ^G^	1.3–2.1 ^G^	6.3–10 ^G^
Triacylglycerides (% fat)	97–98	80–85	97–98
Phospholipids (% fat)	0.5–1.0	0.8–10	0.2–1.0
Free fatty acids (% fat)	0.1–0.2	9.4–9.6	0.1–0.2
Cholesterol (mg/L)	13–26 ^G^	5.0–9.8 ^G^	15–33 ^G^
Fat globule size (μm)	2.8–4.6	2.0–3.0	3.0–3.8
Protein fraction			
Protein content (%)	3.3–3.7 ^G^	2.1–3.9 ^G^	4.5–8.6 ^G^
Casein (% protein)	71–84	49–53 ^G^	75–78 ^G^
α-casein (% casein)	46–50	19–23	30–50
β-casein (% casein)	33–40	79–93	42–62
κ-casein (% casein)	10–12	1.8–2.1	7.5–8.9
Whey protein (% protein)	16–20	36–44 ^G^	14–21 ^G^
α-lactalbumin (% whey protein)	15–24 ^G^	36–38 ^G^	13–24 ^G^
β-lactoglobulin (% whey protein)	64–88 ^G^	29–30 ^G^	64–87 ^G^
Immunoglobulins (% whey protein)	8.0–16 ^G^	15–17 ^G^	4.5–6.6
Lactoferrin (% whey protein)	2.3–2.7 ^G^	7.0–9.2 ^G^	6.4–8.2
Lysozime (% whey protein)	nd	4.4–5.0 ^G^	nd
Micelles size (nm)	150–182	255–312	180–210
Lactose (%)	4.7–5.4 ^G^	6.4–6.7 ^G^	4.1–5.1 ^G^
Ash (%)	0.6–0.8 ^G^	0.3–0.5 ^G^	0.2–0.5 ^G^
Calcium (mg/100 mL or g milk)	119–134 ^G^	85–99 ^G^	55–218 ^G^
Potassium (mg/100 mL or g milk)	135–151 ^G^	54–73 ^G^	104–132 ^G^
Phosphorous (mg/100 mL or g milk)	86–109 ^G^	52–66 ^G^	103–133 ^G^
Ca/P	1.2–1.3	1.5–1.7	1.2–1.3
Vitamins			
Fat soluble vitamins			
Vitamin A (μg/100 mL or g milk)	62–285 ^G^	35–104 ^G^	72–393 ^G^
Vitamin E (μg/100 mL or g milk)	81–166 ^G^	nd-117 ^G^	167–318 ^G^
Water soluble vitamins			
Vitamin C (mg/100 mL or g milk)	0.3–2.3	0.7–8.1	0.4–6.0
Vitamin B1 (μg/100 mL or g milk)	28–90	20–52	28–80
Vitamin B2 (μg/100 mL or g milk)	116–202	5.0–48	160–429
Vitamin B3 (μg/100 mL or g milk)	50–130	70–140	300–500
Vitamin B5 (μg/100 mL or g milk)	260–490	277–300	350–430
Vitamin B6 (μg/100 mL or g milk)	30–70	8.0–61	27–80
Vitamin B9 (μg/100 mL or g milk)	1.0–18	0.13	0.2–6.0
Vitamin B12 (μg/100 mL or g milk)	0.3–0.7	0.3–2.0	0.3–0.7

nd, not detected. ^G^ Data from animals bred under extensive grazing management. Sources: cow [6,23,24,25,26,27,28,29,30,31,32,33,34,35], mare [6,11,13,15,27,28,29,35,36,37,38,39,40,41,42], ewe [6,27,35,43,44,45,46,47,48,49,50,51,52,53,54,55,56,57].

**Table 2:** Comparison of fatty acid profiles (g/100 g total fatty acids) among cow, mare and ewe milk from animals under extensive grazing management.

Fatty Acid	Cow	Mare	Ewe
4:0	0.020–3.3	0.090–0.16	1.9–3.6
6:0	1.1–1.8	0.19–0.39	1.3–2.8
8:0	0.95–2.2	0.58–5.2	1.0–2.7
10:0	1.9–3.7	2.6–11	3.3–7.9
11:0	0.038–0.060	0.030–0.050	0.030–0.33
12:0	2.1–4.1	4.2–9.9	2.4–4.1
13:0	0.070–0.15	0.040–0.19	0.070–0.096
14:0	7.4–12	6.0–9.7	8.5–10
15:0	1.0–2.6	0.22–0.56	0.97–1.2
16:0	19–34	18–27	19–25
17:0	0.53–2.4	0.35–0.53	0.44–0.83
18:0	9.0–17	0.83–4.9	9.3–13
20:0	0.13–0.21	0.080–0.10	0.20–0.36
21:0	0.050–0.49	0.56–0.77	0.010–0.10
22:0	0.060–0.57	0.030–0.30	0.11–0.18
23:0	0.020–0.063	nd	0.064–0.092
24:0	0.040–0.18	nd	0.040–0.080
SFA	52–69	43–58	55–74
10:1	0.24–0.27	1.1–1.7	0.15–0.25
9*c*-12:1	nd–0.080	0.15–0.26	0.030–0.11
9*c*-14:1	0.71–3.4	0.18–0.89	0.14–0.32
9*c*-15:1	0.23–0.27	nd–0.32	0.090–0.14
9*c*-16:1	1.0–3.1	3.2–7.0	0.79–1.3
9*c*-17:1	0.24–1.2	0.27–0.87	0.26–0.38
9*c*-18:1	17–22	14–22	18–21
11*c*-18:1	0.44–0.56	0.71–1.4	0.21–0.29
12*c*-18:1	0.23–0.25	0.69–0.74	0.37–0.46
*t*-18:1	1.3–6.5	nd	2.7–7.0
7*c*-20:1	0.040–1.5	0.25–0.44	0.22–0.32
*11c-22:1*	0.040–0.54	nd	nd
15*c*-24:1	0.010–0.061	nd	nd
MUFA	24–39	18–32	23–31
NC-dienes	0.58–1.5	0.030–0.14	0.88–1.8
CLA	0.49–2.4	0.0010–0.14	1.2–2.8
18:2n-6 (LA)	1.3–3.9	6.2–18	1.7–2.8
18:3n-6	0.040–0.63	0.15–1.3	0.054–0.080
20:2n-6	0.027–0.59	0.12–0.47	0.057–0.070
20:3n-6	0.010–0.11	0.090–0.10	0.030–0.16
20:4n-6	0.040–0.14	0.080–0.60	0.15–0.21
22:2n-6	<0.010–0.14	nd	0.090–0.12
18:3n-3 (LNA)	0.43–1.6	3.7–23	0.82–1.7
20:5n-3 (EPA)	0.060–0.15	nd	0.046–0.17
22:5n-3 (DPA)	0.050–0.10	0.080–0.12	0.11–0.23
22:6n-3 (DHA)	<0.010–0.010	nd	0.020–0.11
PUFA	2.8–7.2	18–31	2.6–7.9

nd, not detected; SFA, saturated fatty acids; c, cis; t, trans; MUFA, monounsaturated fatty acids; NC, non-conjugated; CLA, conjugated linoleic acids; LA, linoleic acid; LNA, linolenic acid; EPA, eicosapentaenoic acid; DPA, docosapentaenoic acid; DHA, docosahexadecanoic acid; PUFA, polyunsaturated fatty acids. Sources: cow [23,24,25,26], mare [36,37,38,39,40], ewe [43,44,45,46,57].

## Data Availability

No new data were created or analyzed in this study. Data sharing is not applicable to this article.
